# Investigating mediated effects of fear of COVID-19 and COVID-19 misunderstanding in the association between problematic social media use, psychological distress, and insomnia

**DOI:** 10.1016/j.invent.2020.100345

**Published:** 2020-08-27

**Authors:** Chung-Ying Lin, Anders Broström, Mark D. Griffiths, Amir H. Pakpour

**Affiliations:** a Department of Rehabilitation Sciences, Faculty of Health & Social Sciences, The Hong Kong Polytechnic University, Hung Hom, Hong Kong; b Department of Nursing, School of Health and Welfare, Jönköping University, Jönköping, Sweden; c Department of Clinical Neurophysiology, University Hospital Linköping, Linköping, Sweden; d Nottingham Trent University, International Gaming Research Unit, Psychology Department, Nottingham, UK; e Social Determinants of Health Research Center, Research Institute for Prevention of Non-Communicable Diseases, Qazvin University of Medical Sciences, Qazvin, Iran

**Keywords:** COVID-19, Social media use, Fear, Iran, Insomnia, Psychological distress

## Abstract

**Introduction:**

Due to the serious situation of the novel coronavirus disease 2019 (COVID-19) worldwide, many countries have implemented policies to minimize the spread of COVID-19 infection. However, some of these policies prevent people from physical contact. Consequently, many individuals may rely on social media to obtain information concerning COVID-19. Unfortunately, social media use (especially problematic social media use) may give rise to psychological distress. Therefore, this study thus examined potential psychopathology to explain the association between problematic social media use, psychological distress, and insomnia.

**Methods:**

Utilizing an online survey, a sample of Iranian young adults (n = 1078 with 628 males; mean age = 26.24 years [SD ± 7.41]) completed questions and psychometric scales concerning psychological distress, insomnia, problematic social media use, fear of COVID-19, and COVID-19 misunderstanding.

**Results:**

Problematic social media use was significantly associated with psychological distress both directly and indirectly. The indirect effects were through fear of COVID-19 (unstandardized coefficient [B] = 0.177; Bootstrapping SE = 0.026) and COVID-19 misunderstanding (B = 0.060; Bootstrapping SE = 0.014). Problematic social media use was significantly associated with insomnia both directly and indirectly. The indirect effect was through fear of COVID-19 (B = 0.062; Bootstrapping SE = 0.019) but not COVID-19 misunderstanding (B = 0.012; Bootstrapping SE = 0.014).

**Discussion/conclusion:**

Due to the pressure of the COVID-19 outbreak, individuals are highly likely to develop psychological distress and insomnia. Apart from developing appropriate health policies to minimize the spread of COVID-19 infection, healthcare providers should design appropriate online campaigns to eliminate people's fear of COVID-19 and to diminish misunderstanding concerning COVID-19.

## Highlights

• Novel coronavirus disease 2019 (COVID-19) causes fear.
• Social media may distribute COVID-19 misinformation and misconceptions.
• Problematic social media associated with distress during COVID-19 outbreak.
• Healthcare providers should design online campaigns to fight misconceptions.

## 1 Introduction

The rapid growth of novel coronavirus disease 2019 (COVID-19) infection spread fast among 213 countries/territories worldwide with over 7.7 million confirmed cases and over 418,000 deaths at the time of writing (June 13, 2020; Worldometer, 2020), and the World Health Organization (WHO) announcing this as a pandemic (World Health Organization, 2020). The threat of COVID-19 has been documented by its high transmission rate (World Health Organization, 2020) and relatively high mortality rate at about 2% (Baud et al., 2020). Although there are some typical COVID-19 symptoms (e.g., fever, fatigue, dry cough, myalgia, and dyspnea), some people infected by COVID-19 may have symptoms similar to influenza (Wang et al., 2020a, Wang et al., 2020b; Wong et al., 2020). Therefore, this may result in some cases of COVID-19 infection being missed and a loophole of COVID-19 infection control. Because of the direct threat to life with a substantial number of confirmed cases, the anxiety of being infected and subsequent sleep problems (e.g., insomnia) have been reported among general population (Wang et al., 2020a, Wang et al., 2020b; Xiao et al., 2020). Using a psychometrically validated instrument on fear of COVID-19, a recent largescale study reported that the Iranian general population had very high fear levels of COVID-19 (Ahorsu et al., 2020b). From past experience, fear may result in negative effects on health for individuals (Lin, 2020; Ren et al., 2020). Therefore, governments need information concerning the fear of COVID-19 among general populations to implement effective policies to control the transmission rate of COVID-19 without increasing psychological distress.

Currently, many governments are paying special attention and effort in an attempt to control COVID-19 infection and have implemented national policies to minimize the spread of the virus (Lin and Cheng, 2020; Rieger, 2020; Shrivastava and Shrivastava, 2020), such as travel and border controls, requirement of citizens to stay at home (California Health and Safety Code, 2020), and prohibition of outdoor activities (Alvarez, 2020). Although such implementation may effectively control the spread of COVID-19, it is highly possible that such implementation also amplifies fear among general population resulting in psychological, social, and economic burdens (Ayittey et al., 2020; Webham et al., 2020). More specifically, these policies force residents to stay indoors and limit physical contact among individuals for substantial amounts of time. With such prolonged stays indoors and without face-to-face contact with family and friends, the human nature of social interaction is violated and has interrupted individuals' daily life routine alongside increased fear of infection, financial stress, and other psychologically stressful factors (Brooks et al., 2020; Lee et al., 2018).

With the prolonged enforced home stay, individuals have no choice but to shift living focus from social activities to indoor activities and may end up engaging more in sedentary behaviors than normal (e.g., internet and social media use; Chen et al., accepted). One reason for increased internet and social media use is that individuals want to obtain information concerning COVID-19. Indeed, a recent Chinese study found that more than 80% of its surveyed 1304 participants reported staying at home 20 to 24 h a day since the outbreak of COVID-19 due to closures of schools and businesses (Wang et al., 2020a, Wang et al., 2020b). The same study also found that more than 90% of the participants obtained COVID-19 information from internet and they were keen to know more about COVID-19, including the COVID-19 transmission route, the medication and vaccine availability and effectiveness, travel advice, overseas COVID-19 control experiences, the number of confirmed cases with locations, COVID-19 prevention advice, tailored-made information for different populations (e.g., children and individuals with chronic illnesses), and detailed information on COVID-19 infection symptoms (Wang et al., 2020a, Wang et al., 2020b). Similar online behavior was observed during the Middle East respiratory syndrome coronavirus (MERS-CoV) outbreak, where disease-related online information searches increased in Korea (Shin et al., 2016).

Unfortunately, disease information obtained from the internet or social media may not always be correct. A recent study assessing knowledge and perceptions of COVID-19 from the general public in the United States and United Kingdom reported that participants believed some misconceptions and falsehoods that had circulated on social media (Geldsetzer, 2020). Moreover, was reported that nearly 200 Iranians died and more than 1000 were poisoned by overconsumption in alcohol because they believed in the rumors on social media claiming that drinking alcohol could cure COVID-19 (Aksut, 2020). The aforementioned tragedy indicates that COVID-19 misunderstanding due to incorrect social media information may lead to psychological distress and inappropriate behaviors. Although numerous mental health professionals and public health experts claim the need to consider psychological distress among different populations during the COVID-19 outbreak (Asmundson and Taylor, 2020; Bao et al., 2020; Lima et al., 2020; Pakpour et al., 2020a, Pakpour et al., 2020b; Shigemura et al., 2020), to the best of the present authors' knowledge, only three studies have collected empirical data examining this issue (Ahorsu et al., 2020a, Ahorsu et al., 2020b; Wang et al., 2020a, Wang et al., 2020b; Xiao et al., 2020). Therefore, collecting empirical data to study psychological distress of a population (such as the young adults in the present sample) during COVID-19 outbreak is vitally important, and will provide meaningful information for healthcare providers to design appropriate campaigns to promote public mental health. More specifically, problematic social media use, fear of COVID-19, COVID-19 misunderstanding, psychological distress, and insomnia are all relevant areas where information should be collected and investigated.

Therefore, the present study thus proposed a potential psychopathology mechanism to explain psychological distress among the Iranian young adults during the COVID-19 public health crisis. It was hypothesized that (i) higher problematic social media use would be associated with the greater fear of COVID-19 and COVID-19 misunderstanding; (ii) greater fear of COVID-19 and COVID-19 misunderstanding would be associated with the higher levels of psychological distress; (iii) greater fear of COVID-19 and COVID-19 misunderstanding would be associated with more severe insomnia; (iv) fear of COVID-19 and COVID-19 misunderstanding would be mediators in the association between problematic social media use and psychological distress; and (v) fear of COVID-19 and COVID-19 misunderstanding would be mediators in the association between problematic social media use and insomnia.

## 2 Methods

### 2.1 Participants and procedure

The study was approved by the Ethics Committee of the Qazvin University of Medical Sciences (IR.QUMS.REC.1398.375). A non-probability sampling strategy was used in the administration of an online survey. Online survey software (*Google Forms*) was utilized to carry out the survey. The study sample was invited via social media, online advertising, community websites, and student's online newsletters. Individuals were eligible to participate if they were 18 years or older, able to read and complete an online consent form and survey in Persian/Farsi, possessing a smartphone, and having access to the internet.

After providing informed consent (online), participants completed online self-report measures of problematic social media use and provided demographic information at an initial data collection stage (T1). One week later, follow-up measures of fear and misunderstanding concerning COVID-19 were completed by the same participants (T2). Finally, two weeks after initial assessment (T3), the same participants were asked to self-report on insomnia and psychological distress.

### 2.2 Measures

#### 2.2.1 Psychological distress

The Hospital Anxiety and Depression Scale (HADS), a 14-item instrument rated on a four-point Likert-type scale (score from 0 to 3), was used to assess the psychological distress. The HADS item scores were summed, with a higher score indicating a greater level of psychological distress. The psychometric properties (including construct validity, concurrent validity, test-retest reliability, and internal consistency) of the Persian HADS have been found satisfactory in prior research (Lin and Pakpour, 2017). The internal consistency of the HADS in the present study was very good (α = 0.81).

#### 2.2.2 Insomnia

The Insomnia Severity Index (ISI), a seven-item scale rated on a five-point Likert-type scale (score from 0 to 4), was used to assess the severity of insomnia. The ISI item scores were summed, with a higher score indicating a greater level of insomnia. The psychometric properties (including construct validity, concurrent validity, test-retest reliability, and internal consistency) of the Persian ISI have been found satisfactory in prior research (Lin et al., 2020). The internal consistency of the ISI in the present study was very good (α = 0.86).

#### 2.2.3 Problematic social media use

The Bergen Social Media Addiction Scale (BSMAS), a six-item instrument rated on a five-point Likert-type scale (score from 1 to 5), was used to assess the severity of problematic social media use. The BSMAS item scores were summed, with a higher score indicating a greater level of being at risk of social media addiction. The psychometric properties (including construct validity, concurrent validity, test-retest reliability, and internal consistency) of the Persian BSMAS have been found satisfactory in prior research (Lin et al., 2017). The internal consistency of the BSMAS in the present study was very good (α = 0.88).

#### 2.2.4 Fear of COVID-19

The Fear of COVID-19 Scale (FCV-19S), a seven-item instrument rated on a five-point Likert-type scale (score from 1 to 5), was used to assess the fear level of COVID-19. The FCV-19S item scores were summed, with a higher score indicating a greater level of COVID-19 fear. The psychometric properties (including construct validity, concurrent validity, test-retest reliability, and internal consistency) of the Persian FCV-19S have been found satisfactory in prior research (Ahorsu et al., 2020b). The internal consistency of the FCV-19S in the present study was very good (α = 0.89).

#### 2.2.5 COVID-19 misunderstanding

Four self-developed items were used to assess how an individual misunderstands COVID-19 information. The four items were “The new coronavirus was deliberately created or released by people”; “A vaccine to cure COVID-19 is available”; “Children/infants and young adults won't get COVID-19”; and “Drinking alcohol can increase COVID-19 risk for me”. The items were rated on a dichotomous scale (true = 1 and false = 0), and a higher score indicated more misunderstanding in COVID-19 information. The internal consistency of the CMS in the present study was very good (Kuder-Richardson Formula 20 = 0.83).

#### 2.2.6 Demographic information

Information regarding the participants' age, gender, educational status, occupational status, and self-reported comorbidities were asked to understand the characteristics of the participants.

### 2.3 Statistical analysis

Participants' demographic information and their scores on HADS, ISI, BSMAS, FCV-19S, and COVID-19 misunderstanding were analyzed using descriptive statistics (i.e., mean [and SD] or frequency [percentage]). Pearson correlations were calculated to understand the relationships between HADS, ISI, BSMAS, FCV-19S, and COVID-19 misunderstanding scores.

**Fig. 1:**
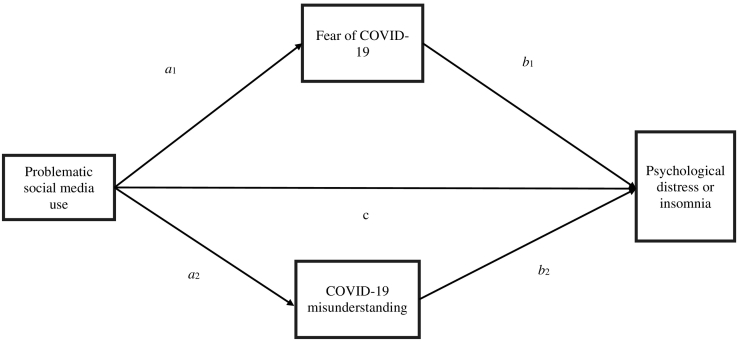
Proposed models that investigate mediated effects in the association between problematic social media use and psychological distress/insomnia. Both models were adjusted for age and gender. Problematic social media use was assessed using Bergen Social Media Addiction Scale; Fear of COVID-19 using Fear of COVID-19 Scale; COVID-19 misunderstanding using four items designed by the authors; psychological distress using Depression, Anxiety, Stress Scale-21; insomnia using Insomnia Severity Index.

Mediation models were analyzed using Model 4 in the Hayes' PROCESS Marco (Hayes, 2018). Two sets of the mediation models were constructed using the same independent variable (i.e., problematic social media use assessed by BSMAS), the same mediators (i.e., fear of COVID-19 assessed using FCV-19S and COVID-19 misunderstanding assessed using the self-developed four items), and the same controlled variables (i.e., age and gender). The only difference between the two mediation models was the different outcomes: the first set included psychological distress (assessed using HADS) as the outcome; the second set included insomnia severity (assessed using ISI) as the outcome (Fig. 1). Both mediation models adopted 10,000 bootstrapping resamples to estimate the mediation effects. All the analyses were performed using SPSS version 24.0 for Windows.

## 3 Results

The sample was relatively young (mean age = 26.24 years [SD ± 7.41]) with fewer males (n = 628; 41.7%). Most of the participants had completed higher education (n = 861; 79.9% with a college or above degree) and had no comorbidities (n = 905; 83.9%) (Table 1). Table 2 reports the mean (and SD) of the participants' levels in psychological distress, problematic social media use, fear of COVID-19, COVID-19 misunderstanding, and insomnia. The aforementioned variables were mutually and significantly correlated (all *p-*values < 0.01). Moreover, psychological distress was moderately correlated with all the other factors (*r* = 0.294 to 0.475) (Table 2).

**Table 1:** Characteristics of the study participants (N = 1078).

	Total mean (±SD) or n (%)	Completers (n = 966)	Non-completers (n = 112)	*p*-Value
Age (year)	26.24 (±7.41)	26.19 (±7.42)	26.67 (±7.36)	0.515
Gender (male)	628 (58.3)	564 (58.4)	64 (57.1)	0.438
Educational status				
Able to read and write	2 (0.2%)	2 (0.2%)	0 (0.0%)	0.702
Primary	9 (0.9%)	7 (0.7%)	2 (1.8%)	
Secondary	107 (9.9%)	96 (9.9%)	11 (9.8%)	
Diplom	99 (9.25)	98 (9.4%)	8 (7.1%)	
College and above	861 (79.9%)	770 (79.9%)	91 (81.3%)	
Occupational status				
Employed	512 (47.5%)	459 (47.5%)	53 (47.3%)	0.525
Unemployed	566 (52.5%)	507 (52.7%)	59 (52.5%)	
Self-reported comorbidities				
No comorbidity	905 (83.9%)	817 (84.6%)	88 (78.6%)	0.101
Diabetes	81 (7.5%)	70 (7.25%)	11 (9.82%)	0.957
Cancer	32 (3.0%)	27 (2.79%)	5 (4.46%)	0.324
Cardiovascular disease	60 (5.6%)	52 (5.38%)	8 (7.14%)	0.441

**Table 2:** Pearson correlation matrix of the variables of interest.

	*r*					Mean (SD)
	1.	2.	3.	4.	5.	
1. Psychological distress^a^	–	0.377	0.475	0.294	0.347	19.16 (7.84)
2. Problematic social media use^b^		–	0.297	0.188	0.142	17.15 (4.86)
3. Fear of COVID-19^c^			–	0.168	0.208	10.28 (4.45)
4. COVID-19 misunderstanding^d^				–	0.098	2.18 (1.02)
5. Insomnia^e^					–	9.25 (5.86)

Note: All *p*-values < 0.01.

a Assessed using Hospital Anxiety and Depression Scale.

b Assessed using Bergen Social Media Addiction Scale.

c Assessed using the Fear of COVID-19 Scale.

d Assessed using a Brief scale on COVID-19 Misunderstanding (appendix).

e Assessed using a Insomnia Severity Index.

Table 3 showed that problematic social media use was significantly associated with psychological distress via both direct (unstandardized coefficient [B] = 0.375; SE = 0.043) and indirect paths. The indirect effects included the path via fear of COVID-19 (B = 0.177; Bootstrapping SE = 0.026) and COVID-19 misunderstanding (B = 0.060; Bootstrapping SE = 0.014). Similarly, problematic social media use was significantly associated with insomnia via both direct (B = 0.095; SE = 0.038) and indirect paths. However, only the indirect path via fear of COVID-19 (B = 0.062; Bootstrapping SE = 0.019) was significant, with the indirect path via COVID-19 misunderstanding (B = 0.012; Bootstrapping SE = 0.014) being non-significant.

**Table 3:** Models that tested mediated effects of fear and misunderstanding.

	Unstand. coeff.	SE (or bootstrapping SE)	*t*-Value (or bootstrapping LLCI)	*p*-Value (or bootstrapping ULCI)
Mediated effects on psychological distress				
Total effect of PSMU on psychological distress	0.611	0.045	13.419	<0.001
Direct effect of PSMU on psychological distress	0.375	0.043	8.736	<0.001
Direct effect of PSMU on mediators				
Fear of COVID-19	0.270	0.027	6.207	<0.001
COVID-19 misunderstanding	0.040	0.006	6.315	<0.001
Indirect effect of PSMU on psychological distress				
Total indirect effect	0.236	(0.030)	(0.181)	(0.299)
Through fear of COVID-19	0.177	(0.026)	(0.127)	(0.230)
Through COVID-19 misunderstanding	0.060	(0.014)	(0.035)	(0.089)

Mediated effects on insomnia				
Total effect of PSMU on insomnia	0.170	0.036	4.664	<0.001
Direct effect of PSMU on insomnia	0.095	0.038	2.512	0.01
Direct effect of PSMU on mediators				
Fear of COVID-19	0.270	0.027	6.202	<0.001
COVID-19 misunderstanding	0.040	0.006	6.313	<0.001
Indirect effect of PSMU on insomnia				
Total indirect effect	0.075	(0.020)	(0.038)	(0.117)
Through fear of COVID-19	0.062	(0.019)	(0.029)	(0.102)
Through COVID-19 misunderstanding	0.012	(0.007)	(−0.001)	(0.029)

Note: Age and gender were adjusted for the model.

Unstand. Coeff. = unstandardized coefficient.

LLCI = lower limit in 95% confidence interval.

ULCI = upper limit in 95% confidence interval.

PSMU = Problematic social media use, assessed using Bergen Social Media Addiction Scale.

## 4 Discussion

The present study is the first to demonstrate the temporal associations between problematic social media use (baseline measures), fear of COVID-19, COVID-19 misunderstanding (one week after baseline measure), psychological distress, and insomnia severity (two weeks after baseline measure). Hayes' mediation model showed that problematic social media use was associated with psychological distress and insomnia both directly and indirectly. More specifically, problematic social media use was indirectly associated with psychological distress via both fear of COVID-19 and COVID-19 misunderstanding; problematic social media use was indirectly associated with insomnia only via fear of COVID-19. Moreover, higher levels of problematic social media use were associated with greater fear of COVID-19 and more misunderstanding of COVID-19.

Because the mortality rate of COVID-19 is not low and the individuals recovered from COVID-19 are likely to have health consequences (Baud et al., 2020), general populations have reason to feel fear of COVID19 (Wang et al., 2020a, Wang et al., 2020b; Xiao et al., 2020). However, the present study showed that such fear may be magnified when the individuals received negative COVID-19 information from social media. From the present findings, higher levels of problematic social media use were associated with a higher level of fear of COVID-19 one week later. Individuals with greater problematic social media use are likely to be exposed more to COVID-19 information than those with less problematic social media use. Given that a substantial amount of misinformation and misconception can be found in social media (Geldsetzer, 2020), individuals with greater problematic social media use are therefore more likely to have greater COVID-19 misunderstanding.

The fear of COVID-19 and COVID-19 misunderstanding were found to be associated with psychological distress in the present study. This finding was expected because fear is usually an initial psychological response for individuals to respond to threat, and this can allow the individuals take action to protect themselves (Lang et al., 2000). However, when an individual cannot tackle the fear for a specific period of time, prolonged fear may subsequently lead to the development other types of psychological distress, such anxiety and depression (Kuriyama et al., 2010). This human biopsychological mechanism also helps explain why fear of COVID-19 appeared to be associated with the development of psychological distress in the present study's sample. Regarding COVID-19 misunderstanding, it is likely that when individuals receive substantial amounts of misinformation and misconceptions concerning COVID-19 (Geldsetzer, 2020), their uneasiness is increased and consequently reflected by psychological distress.

The fear of COVID-19 but not COVID-19 misunderstanding was found to be a factor that was associated with insomnia in the present study. With substantial evidence showing that individuals with fear have sleep problems (Kuriyama et al., 2010), it is reasonable that fear of COVID-19 was associated with insomnia. That is, when an individual worries and fears COVID-19, the individual's brain is stimulated and excited (Kuriyama et al., 2010). Therefore, the individual cannot take rest and develops insomnia. This finding may supplement the finding from Xiao et al. (2020) that individuals during the COVID-19 pandemic have sleep problems. Although COVID-19 misunderstanding was not directly associated with insomnia, COVID-19 misunderstanding may be indirectly associated with insomnia via psychological distress. More specifically, psychological distress was found to be associated with insomnia in previous research (Belleville and Morin, 2008). Therefore, diminishing COVID-19 misunderstanding may also help overcome insomnia among the general population. However, stronger evidence is needed concerning causal relationships between the variables examined in the present study.

There are some limitations in the present study. First, it is unclear how, where, and what COVID-19 information the participants obtained. Moreover, the items used in the present study to assess COVID-19 misunderstanding did not cover all the misconceptions on COVID-19. Therefore, impacts of specific misinformation cannot be determined and the COVID-19 misunderstanding found in the present study may be underestimated. Second, a convenience sampling utilizing an online survey was used to collect data among Iranians. Therefore, the generalizability of the present study's findings is restricted (i.e., the present study's sample does not represent all Iranian young adults and the study's results cannot necessarily be generalized to Western populations). Third, although the present study intended to collect longitudinal data across three time points with an interval of one week apart, the temporal effects might be trivial because it is hard for an individual to change the mental capacity in such short time periods. However, in order to provide immediate and timely information on psychological distress during the COVID-19 pandemic, the present authors reasoned that the optimal method to understand temporal effects was to use short interval periods. Nevertheless, the present study cannot provide strong evidence in terms of causality given the following issues: (i) the lack of information on potential confounders (i.e., financial status and wellbeing, changes in living arrangements, changes in lifestyle) and (ii) the lack of baseline assessments concerning psychological distress and insomnia. Finally, all the data were self-report and the common method biases from collecting such data cannot be avoided. For example, participants may have responded with socially desirable answers and reported less problematic social media use and better health.

## 5 Conclusions

Due to the pressure of the COVID-19 outbreak, general populations are highly likely to develop psychological distress and insomnia. The elevated psychological distress may be triggered by fear of COVID-19 and COVID-19 misunderstanding. Insomnia may be induced by fear of COVID-19. Therefore, in addition to developing appropriate health and social policies to help minimize the spread of COVID-19, healthcare providers should also design appropriate online campaigns to help minimize the general population's fear of COVID-19 and to diminish the COVID-19 misunderstanding. More specifically, healthcare providers may encourage individuals to limit their social media use more generally (especially given the amount of social media featuring wrong or fake information) during the times of crisis. Instead, healthcare providers should provide trusted sources of news for the general population in obtaining accurate information.

## Statement of authorship

Participants' recruitment: A. H. Pakpour.

Data acquisition and analysis: A. H. Pakpour.

Writing Original draft: C.-Y. Lin.

Critically reviewing draft: A. Broström and M. D. Griffiths.

Supervision: A. H. Pakpour.

## Funding sources

None.

## Declaration of competing interest

The authors of this paper declare that there are not any conflict of interest.
